# Supplementary material for: Modelling Skylarks (Alauda arvensis) to Predict Impacts of Changes in Land Management and Policy: Development and Testing of an Agent-Based Model
Source: PLoS One. 2013 Jun 6;8(6):e65803. doi: 10.1371/journal.pone.0065803 (PMC3675089; doi:10.1371/journal.pone.0065803)
Supplement: Supporting Information S4 — The skylark ODdox as a zipped archive. (ZIP) [file pone.0065803.s004.zip › Skylark_ODdox/elements_8cpp.html]

ALMaSS Skylark ODdox: elements.cpp File Reference


ALMaSS Skylark ODdox
 2.0


- Main Page
- Related Pages
- Classes
- Files

- File List
- File Members

Macros |
Functions |
Variables

elements.cpp File Reference

`#include <math.h>`  
`#include "../Landscape/ls.h"`

|  |  |
| --- | --- |
| Macros | |
| #define | \_\_WEED\_CURVE   99 |
| #define | \_CRT\_SECURE\_NO\_DEPRECATE |
| #define | EL\_BUG\_GLUE   (l\_el\_bug\_glue.value()) |
| #define | EL\_BUG\_PERCENT\_A   0.0 |
| #define | EL\_BUG\_PERCENT\_B   0.2975 |
| #define | EL\_BUG\_PERCENT\_C   0.095916647275 |
| #define | EL\_BUG\_PERCENT\_D   0 |
| #define | EL\_BUG\_PERCENT\_D   0 |
| #define | EL\_BUG\_PERCENT\_D   0 |
| #define | EL\_BUG\_PERCENT\_D   0 |
| #define | EL\_BUG\_PERCENT\_D   0 |
| #define | EL\_BUG\_PERCENT\_D   0 |
| #define | EL\_BUG\_PERCENT\_D   0 |
| #define | EL\_BUG\_PERCENT\_Edges\_A   10.72459109 |
| #define | EL\_BUG\_PERCENT\_Edges\_B   0.4 |
| #define | EL\_BUG\_PERCENT\_Edges\_C   2.529631141 |
| #define | EL\_BUG\_PERCENT\_G\_A   4.123817127 |
| #define | EL\_BUG\_PERCENT\_G\_B   0.151015629 |
| #define | EL\_BUG\_PERCENT\_G\_C   -0.228228353 |
| #define | EL\_BUG\_PERCENT\_SB\_A   0 |
| #define | EL\_BUG\_PERCENT\_SB\_B   0.380763296 |
| #define | EL\_BUG\_PERCENT\_SB\_C   0 |
| #define | EL\_BUG\_PERCENT\_WR\_A   0.0 |
| #define | EL\_BUG\_PERCENT\_WR\_B   0.028271643 |
| #define | EL\_BUG\_PERCENT\_WR\_C   0.0 |
| #define | EL\_BUG\_PERCENT\_WRy\_A   0.0 |
| #define | EL\_BUG\_PERCENT\_WRy\_B   0.395651915 |
| #define | EL\_BUG\_PERCENT\_WRy\_C   0.0 |
| #define | EL\_BUG\_PERCENT\_WW\_A   0.0 |
| #define | EL\_BUG\_PERCENT\_WW\_B   0.1283 |
| #define | EL\_BUG\_PERCENT\_WW\_C   0.0 |
| #define | EL\_BUG\_SLOPE   (l\_el\_bug\_slope.value()) |
| #define | EL\_GROWTH\_DATE\_MAGIC   (g\_date->DayInYear(1,9)) |
| #define | EL\_GROWTH\_DAYDEG\_MAGIC   l\_el\_growth\_daydeg\_magic.value() |
| #define | EL\_GROWTH\_PHASE\_SHIFT\_LEVEL   (l\_el\_growth\_phase\_shift\_level.value()) |
| #define | EL\_PLANT\_BIOMASS   (l\_el\_plant\_biomass\_proport.value()) |
| #define | EL\_VEG\_HEIGHTSCALE   (l\_el\_veg\_heightscale.value()) |
| #define | EL\_VEG\_START\_LAIT   (l\_el\_veg\_start\_lait.value()) |
| #define | EL\_WEED\_GLUE   (l\_el\_weed\_glue.value()) |
| #define | EL\_WEED\_PERCENT   (l\_el\_weed\_percent.value()) |
| #define | EL\_WEED\_SLOPE   (l\_el\_weed\_slope.value()) |
| #define | RV\_CUT\_AUG   (l\_el\_rv\_cut\_aug.value()) |
| #define | RV\_CUT\_GREEN   (l\_el\_rv\_cut\_green.value()) |
| #define | RV\_CUT\_HEIGHT   (l\_el\_rv\_cut\_height.value()) |
| #define | RV\_CUT\_JUL   (l\_el\_rv\_cut\_jul.value()) |
| #define | RV\_CUT\_JUN   (l\_el\_rv\_cut\_jun.value()) |
| #define | RV\_CUT\_MAY   (l\_el\_rv\_cut\_may.value()) |
| #define | RV\_CUT\_OCT   (l\_el\_rv\_cut\_oct.value()) |
| #define | RV\_CUT\_SEP   (l\_el\_rv\_cut\_sep.value()) |
| #define | RV\_CUT\_TOTAL   (l\_el\_rv\_cut\_total.value()) |
| #define | RV\_MAY\_1ST   (l\_el\_rv\_may\_1st.value()) |

|  |  |
| --- | --- |
| Functions | |
| void | FloatToDouble (double &, float) |

|  |  |
| --- | --- |
| Variables | |
| const double | c\_SolarConversion [2][81] |
| static CfgFloat | cfg\_beetlebankinsectscaler ("ELE\_BBINSECTSCALER", CFG\_CUSTOM, 1.0) |
| static CfgFloat | cfg\_ele\_weedscaling ("ELE\_WEEDSCALING", CFG\_CUSTOM, 1.0) |
| static CfgInt | cfg\_OrchardNoCutsDay ("TOX\_ORCHARDNOCUTS", CFG\_CUSTOM,-1) |
| static CfgInt | cfg\_OrchardSprayDay ("TOX\_ORCHARDSPRAYDAY", CFG\_CUSTOM, 150) |
| static CfgInt | cfg\_OrchardSprayDay2 ("TOX\_ORCHARDSPRAYDAYTWO", CFG\_CUSTOM, 200000) |
| CfgInt | cfg\_pest\_productapplic\_period |
| CfgInt | cfg\_pest\_productapplic\_startdate |
| CfgInt | cfg\_pest\_productapplic\_startdate2 |
| static CfgInt | cfg\_UMPatchyChance ("UMPATCHYCHANCE", CFG\_CUSTOM, 50) |
| static double | g\_biomass\_scale [tov\_Undefined] |
| static double | g\_bug\_percent\_a [tov\_Undefined] |
| static double | g\_bug\_percent\_b [tov\_Undefined] |
| static double | g\_bug\_percent\_c [tov\_Undefined] |
| static double | g\_bug\_percent\_d [tov\_Undefined] |
| CfgInt | g\_el\_herbicide\_delaytime\_days ("ELEM\_HERBICIDE\_DELAYTIME\_DAYS", CFG\_PRIVATE, 35) |
| CfgInt | g\_el\_strigling\_delaytime\_days ("ELEM\_STRIGLING\_DELAYTIME\_DAYS", CFG\_PRIVATE, 28) |
| CfgInt | g\_el\_tramline\_decaytime\_days ("ELEM\_TRAMLINE\_DECAYTIME\_DAYS", CFG\_PRIVATE, 21) |
| class LE\_TypeClass \* | g\_letype |
| static double | g\_weed\_percent [tov\_Undefined] |
| static CfgFloat | l\_el\_bug\_glue ("ELEM\_BUG\_GLUE", CFG\_PRIVATE, 0.50) |
| static CfgFloat | l\_el\_bug\_slope ("ELEM\_BUG\_SLOPE", CFG\_PRIVATE, 0.2) |
| static CfgInt | l\_el\_growth\_daydeg\_magic ("ELEM\_GROWTH\_DAYDEG\_MAGIC", CFG\_PRIVATE, 100) |
| static CfgFloat | l\_el\_growth\_phase\_shift\_level ("ELEM\_GROWTH\_PHASE\_SHIFT\_LEVEL", CFG\_PRIVATE, 0.5) |
| static CfgFloat | l\_el\_o\_cut\_green ("ELEM\_RV\_CUT\_GREEN", CFG\_PRIVATE, 1.5) |
| static CfgFloat | l\_el\_o\_cut\_height ("ELEM\_RV\_CUT\_HEIGHT", CFG\_PRIVATE, 10.0) |
| static CfgFloat | l\_el\_o\_cut\_total ("ELEM\_RV\_CUT\_TOTAL", CFG\_PRIVATE, 2.0) |
| static CfgFloat | l\_el\_plant\_biomass\_proport ("ELEM\_PLANT\_BIOMASS\_PROPORT", CFG\_PRIVATE, 41.45) |
| static CfgInt | l\_el\_rv\_cut\_aug ("ELEM\_RV\_CUT\_AUG", CFG\_PRIVATE, 42) |
| static CfgFloat | l\_el\_rv\_cut\_green ("ELEM\_RV\_CUT\_GREEN", CFG\_PRIVATE, 1.5) |
| static CfgFloat | l\_el\_rv\_cut\_height ("ELEM\_RV\_CUT\_HEIGHT", CFG\_PRIVATE, 10.0) |
| static CfgInt | l\_el\_rv\_cut\_jul ("ELEM\_RV\_CUT\_JUL", CFG\_PRIVATE, 35) |
| static CfgInt | l\_el\_rv\_cut\_jun ("ELEM\_RV\_CUT\_JUN", CFG\_PRIVATE, 28) |
| static CfgInt | l\_el\_rv\_cut\_may ("ELEM\_RV\_CUT\_MAY", CFG\_PRIVATE, 142) |
| static CfgInt | l\_el\_rv\_cut\_oct ("ELEM\_RV\_CUT\_OCT", CFG\_PRIVATE, 49) |
| static CfgInt | l\_el\_rv\_cut\_sep ("ELEM\_RV\_CUT\_SEP", CFG\_PRIVATE, 49) |
| static CfgFloat | l\_el\_rv\_cut\_total ("ELEM\_RV\_CUT\_TOTAL", CFG\_PRIVATE, 2.0) |
| static CfgInt | l\_el\_rv\_may\_1st ("ELEM\_RV\_MAY\_1ST", CFG\_PRIVATE, 121) |
| static CfgInt | l\_el\_veg\_heightscale ("ELEM\_VEG\_HEIGHTSCALE", CFG\_PRIVATE, 16) |
| static CfgFloat | l\_el\_veg\_start\_lait ("ELEM\_VEG\_START\_LAIT", CFG\_PRIVATE, 1.08) |
| static CfgFloat | l\_el\_weed\_glue ("ELEM\_WEED\_GLUE", CFG\_PRIVATE, 0.99) |
| static CfgFloat | l\_el\_weed\_percent ("ELEM\_WEED\_PERCENT", CFG\_PRIVATE, 0.1) |
| static CfgFloat | l\_el\_weed\_slope ("ELEM\_WEED\_SLOPE", CFG\_PRIVATE, 0.15) |
| CfgFloat | l\_pest\_insecticide\_amount |
| static CfgFloat | l\_pest\_productOrchard\_amount ("PEST\_PRODUCTORCHARD\_AMOUNT", CFG\_CUSTOM, 0.0) |

---

## Macro Definition Documentation

#define \_\_WEED\_CURVE   99

#define \_CRT\_SECURE\_NO\_DEPRECATE

#define EL\_BUG\_GLUE   (l\_el\_bug\_glue.value())

#define EL\_BUG\_PERCENT\_A   0.0

Referenced by VegElement::VegElement().

#define EL\_BUG\_PERCENT\_B   0.2975

Referenced by VegElement::VegElement().

#define EL\_BUG\_PERCENT\_C   0.095916647275

Referenced by VegElement::VegElement().

#define EL\_BUG\_PERCENT\_D   0

Referenced by VegElement::VegElement().

#define EL\_BUG\_PERCENT\_D   0

#define EL\_BUG\_PERCENT\_D   0

#define EL\_BUG\_PERCENT\_D   0

#define EL\_BUG\_PERCENT\_D   0

#define EL\_BUG\_PERCENT\_D   0

#define EL\_BUG\_PERCENT\_D   0

#define EL\_BUG\_PERCENT\_Edges\_A   10.72459109

Referenced by VegElement::VegElement().

#define EL\_BUG\_PERCENT\_Edges\_B   0.4

Referenced by VegElement::VegElement().

#define EL\_BUG\_PERCENT\_Edges\_C   2.529631141

Referenced by VegElement::VegElement().

#define EL\_BUG\_PERCENT\_G\_A   4.123817127

Referenced by VegElement::VegElement().

#define EL\_BUG\_PERCENT\_G\_B   0.151015629

Referenced by VegElement::VegElement().

#define EL\_BUG\_PERCENT\_G\_C   -0.228228353

Referenced by VegElement::VegElement().

#define EL\_BUG\_PERCENT\_SB\_A   0

Referenced by VegElement::VegElement().

#define EL\_BUG\_PERCENT\_SB\_B   0.380763296

Referenced by VegElement::VegElement().

#define EL\_BUG\_PERCENT\_SB\_C   0

Referenced by VegElement::VegElement().

#define EL\_BUG\_PERCENT\_WR\_A   0.0

Referenced by VegElement::VegElement().

#define EL\_BUG\_PERCENT\_WR\_B   0.028271643

Referenced by VegElement::VegElement().

#define EL\_BUG\_PERCENT\_WR\_C   0.0

Referenced by VegElement::VegElement().

#define EL\_BUG\_PERCENT\_WRy\_A   0.0

Referenced by VegElement::VegElement().

#define EL\_BUG\_PERCENT\_WRy\_B   0.395651915

Referenced by VegElement::VegElement().

#define EL\_BUG\_PERCENT\_WRy\_C   0.0

Referenced by VegElement::VegElement().

#define EL\_BUG\_PERCENT\_WW\_A   0.0

Referenced by VegElement::VegElement().

#define EL\_BUG\_PERCENT\_WW\_B   0.1283

Referenced by VegElement::VegElement().

#define EL\_BUG\_PERCENT\_WW\_C   0.0

Referenced by VegElement::VegElement().

#define EL\_BUG\_SLOPE   (l\_el\_bug\_slope.value())

#define EL\_GROWTH\_DATE\_MAGIC   (g\_date->DayInYear(1,9))

Referenced by VegElement::ReduceVeg\_Extended().

#define EL\_GROWTH\_DAYDEG\_MAGIC   l\_el\_growth\_daydeg\_magic.value()

Referenced by VegElement::ReduceVeg\_Extended().

#define EL\_GROWTH\_PHASE\_SHIFT\_LEVEL   (l\_el\_growth\_phase\_shift\_level.value())

Referenced by VegElement::ReduceVeg\_Extended().

#define EL\_PLANT\_BIOMASS   (l\_el\_plant\_biomass\_proport.value())

Referenced by UnsprayedFieldMargin::DoDevelopment().

#define EL\_VEG\_HEIGHTSCALE   (l\_el\_veg\_heightscale.value())

Referenced by VegElement::RandomVegStartValues().

#define EL\_VEG\_START\_LAIT   (l\_el\_veg\_start\_lait.value())

Referenced by VegElement::RandomVegStartValues().

#define EL\_WEED\_GLUE   (l\_el\_weed\_glue.value())

#define EL\_WEED\_PERCENT   (l\_el\_weed\_percent.value())

#define EL\_WEED\_SLOPE   (l\_el\_weed\_slope.value())

#define RV\_CUT\_AUG   (l\_el\_rv\_cut\_aug.value())

Referenced by RoadsideVerge::DoDevelopment().

#define RV\_CUT\_GREEN   (l\_el\_rv\_cut\_green.value())

Referenced by RoadsideVerge::Cutting().

#define RV\_CUT\_HEIGHT   (l\_el\_rv\_cut\_height.value())

Referenced by RoadsideVerge::Cutting().

#define RV\_CUT\_JUL   (l\_el\_rv\_cut\_jul.value())

Referenced by RoadsideVerge::DoDevelopment().

#define RV\_CUT\_JUN   (l\_el\_rv\_cut\_jun.value())

Referenced by RoadsideVerge::DoDevelopment().

#define RV\_CUT\_MAY   (l\_el\_rv\_cut\_may.value())

Referenced by RoadsideVerge::DoDevelopment().

#define RV\_CUT\_OCT   (l\_el\_rv\_cut\_oct.value())

Referenced by RoadsideVerge::DoDevelopment().

#define RV\_CUT\_SEP   (l\_el\_rv\_cut\_sep.value())

Referenced by RoadsideVerge::DoDevelopment().

#define RV\_CUT\_TOTAL   (l\_el\_rv\_cut\_total.value())

Referenced by RoadsideVerge::Cutting().

#define RV\_MAY\_1ST   (l\_el\_rv\_may\_1st.value())

Referenced by RoadsideVerge::DoDevelopment().

---

## Function Documentation

|  |  |  |  |
| --- | --- | --- | --- |
| void FloatToDouble | ( | double & | , |
|  |  | float |  |
|  | ) |  |  |

{

char \* num = 0;

num = new char[\_CVTBUFSIZE];

errno\_t err = \_gcvt\_s(num, \_CVTBUFSIZE,f,8);

if (err!=0) {

assert(0);

}

d = atof(num);

delete [] num;

}

---

## Variable Documentation

const double c\_SolarConversion[2][81]

**Initial value:**

{

{

0,0,0,0,0,0,0,0,0,0,0,0,0,0,0,0,0,0,0,0,0,0,0,0,0,0,0,0,0,0,0,0,0,0,0,0.28,

0.56,0.84,1.12,1.4,1.4,1.4,1.4,1.4,1.4,1.4,1.4,1.4,1.4,1.4,1.4,1.4,1.4,1.4,

1.4,1.4,1.26,1.12,0.98,0.84,0.7,0.56,0.42,0.28,0.14,0,0,0,0,0,0,0,0,0,0,0,

0,0,0,0,0

},

{

0,0,0,0,0,0,0,0,0,0,0,0,0,0,0,0,0,0,0,0,0,0,0,0,0,0,0,0,0,0,0,0,0,0,0,0,0,

0,0.242857,0.485714,0.728571,0.971429,1.214286,1.457143,1.7,1.7,1.7,1.7,

1.7,1.7,1.7,1.7,1.7,1.7,1.7,1.7,1.7,1.7,1.7,1.7,1.7,1.7,1.7,1.7,1.7,1.7,

1.53,1.36,1.19,1.02,0.85,0.68,0.51,0.34,0.17,0,0,0,0,0,0

}

}

Referenced by VegElement::RecalculateBugsNStuff().

CfgFloat cfg\_beetlebankinsectscaler("ELE\_BBINSECTSCALER", CFG\_CUSTOM, 1.0)

static

Referenced by BeetleBank::DoDevelopment().

CfgFloat cfg\_ele\_weedscaling("ELE\_WEEDSCALING", CFG\_CUSTOM, 1.0)

static

Referenced by VegElement::DoDevelopment().

CfgInt cfg\_OrchardNoCutsDay("TOX\_ORCHARDNOCUTS", CFG\_CUSTOM,-1)

static

Referenced by Orchard::DoDevelopment(), and OrchardGrass::DoDevelopment().

CfgInt cfg\_OrchardSprayDay("TOX\_ORCHARDSPRAYDAY", CFG\_CUSTOM, 150)

static

CfgInt cfg\_OrchardSprayDay2("TOX\_ORCHARDSPRAYDAYTWO", CFG\_CUSTOM, 200000)

static

CfgInt cfg\_pest\_productapplic\_period

CfgInt cfg\_pest\_productapplic\_startdate

CfgInt cfg\_pest\_productapplic\_startdate2

Referenced by Orchard::DoDevelopment(), and OrchardBand::DoDevelopment().

CfgInt cfg\_UMPatchyChance("UMPATCHYCHANCE", CFG\_CUSTOM, 50)

static

Referenced by UnsprayedFieldMargin::UnsprayedFieldMargin().

double g\_biomass\_scale[tov\_Undefined]

static

Referenced by UnsprayedFieldMargin::DoDevelopment(), VegElement::RecalculateBugsNStuff(), and VegElement::VegElement().

double g\_bug\_percent\_a[tov\_Undefined]

static

Referenced by UnsprayedFieldMargin::DoDevelopment(), VegElement::ReadBugPercentageFile(), VegElement::RecalculateBugsNStuff(), and VegElement::VegElement().

double g\_bug\_percent\_b[tov\_Undefined]

static

Referenced by UnsprayedFieldMargin::DoDevelopment(), VegElement::ReadBugPercentageFile(), VegElement::RecalculateBugsNStuff(), and VegElement::VegElement().

double g\_bug\_percent\_c[tov\_Undefined]

static

Referenced by UnsprayedFieldMargin::DoDevelopment(), VegElement::ReadBugPercentageFile(), VegElement::RecalculateBugsNStuff(), and VegElement::VegElement().

double g\_bug\_percent\_d[tov\_Undefined]

static

Referenced by UnsprayedFieldMargin::DoDevelopment(), VegElement::ReadBugPercentageFile(), and VegElement::VegElement().

CfgInt g\_el\_herbicide\_delaytime\_days("ELEM\_HERBICIDE\_DELAYTIME\_DAYS", CFG\_PRIVATE, 35)

CfgInt g\_el\_strigling\_delaytime\_days("ELEM\_STRIGLING\_DELAYTIME\_DAYS", CFG\_PRIVATE, 28)

CfgInt g\_el\_tramline\_decaytime\_days("ELEM\_TRAMLINE\_DECAYTIME\_DAYS", CFG\_PRIVATE, 21)

class LE\_TypeClass\* g\_letype

Referenced by Landscape::BackTranslateEleTypes(), Landscape::BackTranslateVegTypes(), Farm::HandleEvents(), Farm::InitiateManagement(), Landscape::Landscape(), Landscape::ReadPolys(), Landscape::TranslateEleTypes(), Landscape::TranslateVegTypes(), and Landscape::~Landscape().

double g\_weed\_percent[tov\_Undefined]

static

Referenced by VegElement::ReadBugPercentageFile(), and VegElement::VegElement().

CfgFloat l\_el\_bug\_glue("ELEM\_BUG\_GLUE", CFG\_PRIVATE, 0.50)

static

CfgFloat l\_el\_bug\_slope("ELEM\_BUG\_SLOPE", CFG\_PRIVATE, 0.2)

static

CfgInt l\_el\_growth\_daydeg\_magic("ELEM\_GROWTH\_DAYDEG\_MAGIC", CFG\_PRIVATE, 100)

static

CfgFloat l\_el\_growth\_phase\_shift\_level("ELEM\_GROWTH\_PHASE\_SHIFT\_LEVEL", CFG\_PRIVATE, 0.5)

static

CfgFloat l\_el\_o\_cut\_green("ELEM\_RV\_CUT\_GREEN", CFG\_PRIVATE, 1.5)

static

Referenced by Orchard::Cutting(), and OrchardGrass::Cutting().

CfgFloat l\_el\_o\_cut\_height("ELEM\_RV\_CUT\_HEIGHT", CFG\_PRIVATE, 10.0)

static

Referenced by Orchard::Cutting(), and OrchardGrass::Cutting().

CfgFloat l\_el\_o\_cut\_total("ELEM\_RV\_CUT\_TOTAL", CFG\_PRIVATE, 2.0)

static

Referenced by Orchard::Cutting(), and OrchardGrass::Cutting().

CfgFloat l\_el\_plant\_biomass\_proport("ELEM\_PLANT\_BIOMASS\_PROPORT", CFG\_PRIVATE, 41.45)

static

CfgInt l\_el\_rv\_cut\_aug("ELEM\_RV\_CUT\_AUG", CFG\_PRIVATE, 42)

static

CfgFloat l\_el\_rv\_cut\_green("ELEM\_RV\_CUT\_GREEN", CFG\_PRIVATE, 1.5)

static

CfgFloat l\_el\_rv\_cut\_height("ELEM\_RV\_CUT\_HEIGHT", CFG\_PRIVATE, 10.0)

static

CfgInt l\_el\_rv\_cut\_jul("ELEM\_RV\_CUT\_JUL", CFG\_PRIVATE, 35)

static

CfgInt l\_el\_rv\_cut\_jun("ELEM\_RV\_CUT\_JUN", CFG\_PRIVATE, 28)

static

CfgInt l\_el\_rv\_cut\_may("ELEM\_RV\_CUT\_MAY", CFG\_PRIVATE, 142)

static

CfgInt l\_el\_rv\_cut\_oct("ELEM\_RV\_CUT\_OCT", CFG\_PRIVATE, 49)

static

CfgInt l\_el\_rv\_cut\_sep("ELEM\_RV\_CUT\_SEP", CFG\_PRIVATE, 49)

static

CfgFloat l\_el\_rv\_cut\_total("ELEM\_RV\_CUT\_TOTAL", CFG\_PRIVATE, 2.0)

static

CfgInt l\_el\_rv\_may\_1st("ELEM\_RV\_MAY\_1ST", CFG\_PRIVATE, 121)

static

CfgInt l\_el\_veg\_heightscale("ELEM\_VEG\_HEIGHTSCALE", CFG\_PRIVATE, 16)

static

CfgFloat l\_el\_veg\_start\_lait("ELEM\_VEG\_START\_LAIT", CFG\_PRIVATE, 1.08)

static

CfgFloat l\_el\_weed\_glue("ELEM\_WEED\_GLUE", CFG\_PRIVATE, 0.99)

static

CfgFloat l\_el\_weed\_percent("ELEM\_WEED\_PERCENT", CFG\_PRIVATE, 0.1)

static

CfgFloat l\_el\_weed\_slope("ELEM\_WEED\_SLOPE", CFG\_PRIVATE, 0.15)

static

CfgFloat l\_pest\_insecticide\_amount

CfgFloat l\_pest\_productOrchard\_amount("PEST\_PRODUCTORCHARD\_AMOUNT", CFG\_CUSTOM, 0.0)

static

Referenced by Orchard::DoDevelopment(), and OrchardBand::DoDevelopment().


- CJT
- MSVC
- ALMaSS Working Source
- Landscape
- elements.cpp
- Generated on Thu Jan 10 2013 13:15:35 for ALMaSS Skylark ODdox by
   1.8.1.1
